# Contribution of arbuscular mycorrhiza and exoenzymes to nitrogen acquisition of sorghum under drought

**DOI:** 10.3389/fpls.2025.1514416

**Published:** 2025-04-15

**Authors:** Rosepiah Munene, Osman Mustafa, Sara Loftus, Callum C. Banfield, Reimund P. Rötter, Ezekiel K. Bore, Benard Mweu, Kevin Z. Mganga, Dennis O. Otieno, Mutez A. Ahmed, Michaela A. Dippold

**Affiliations:** ^1^ Biogeochemistry of Agroecosystems, Department of Crop Sciences, University of Goettingen, Goettingen, Germany; ^2^ Geo-Biosphere Interactions, Department of Geosciences, University of Tuebingen, Tuebingen, Germany; ^3^ Institute of Bio- and Geosciences IBG-3, Agrosphere, Juelich Research Center, Juelich, Germany; ^4^ Department of Botany and Agricultural Biotechnology, University of Khartoum, Khartoum, Sudan; ^5^ Tropical Plant Production and Agricultural Systems Modelling (TROPAGS), University of Goettingen, Goettingen, Germany; ^6^ Centre for Biodiversity and Sustainable Land Use (CBL), University of Goettingen, Goettingen, Germany; ^7^ Environmnetal Soil Science, Department of Agricultural Sciences, University of Helsinki, Helsinki, Finland; ^8^ School of Agriculture, Environment, Water and Natural Resources, South Eastern Kenya University, Kitui, Kenya; ^9^ Copernicus Institute of Sustainable Development, Utrecht University, Utrecht, Netherlands; ^10^ School of Biological and Physical Sciences, Jaramogi Oginga Odinga University of Science & Technology (JOOUST), Bondo, Kenya; ^11^ Root-Soil Interaction, TUM School of Life Sciences, Technical University of Munich, Freising, Germany

**Keywords:** enzyme activity, mycorrhiza, photoassimilate use, moisture limitation, nitrogen mobilization, sorghum

## Abstract

**Introduction:**

For low-fertile and degraded soils of sub-Saharan Africa, nitrogen (N) is often the most growth-limiting factor restricting crop yields. The often-suggested exploitation of advantageous rhizosphere traits such as enzyme secretion and/or the symbiosis with arbuscular mycorrhizal fungi (AMF) remains to be validated as a potential strategy to overcome N limitation, especially when N deficiency co-occurs with further abiotic stresses such as water scarcity.

**Methods:**

Three sorghum genotypes were cultivated in soil mesocosms with a root-exclusion compartment, where only AMF could scavenge for nutrients under drought and optimal conditions. Plant carbon (C) investment into the rhizosphere and N uptake were tracked by ^15^N application coupled with ^13^CO_2_ labeling

**Results:**

Under drought, uptake of mineral ^15^N by AMF from the root-exclusion compartment increased 4–12 times compared to well-watered conditions. In addition, water stress enhanced below-ground allocation of recently assimilated C into microbial biomass. Drought reduced the enzymatic potential (Vmax) of chitinase while increasing leucine aminopeptidase (LAP) activity. This suggests that N acquisition via protein mineralization in soil was relatively enhanced compared to that of chitin following moisture limitation. LAP substrate affinity (Km) was reduced by drought compared to that of chitinase with genotype-specific shifts in the rhizosphere enzyme systems observed.

**Conclusion:**

Our findings suggest that below-ground C allocation activated AMF symbiosis and its associated microbiome. This not only led to a shift in enzyme-driven exploitation of distinct organic N sources but also induced a strong increase in AMF-based mineral N acquisition from the mycosphere. This trait plasticity in response to drought may be harnessed to stabilize food production from low-fertile soil under the increasingly negative impacts of droughts due to climate change.

## 1 Introduction

The latest climate change models project continued warming and a decline in annual precipitation accompanied by frequent and severe drought events in many regions of the world, including sub-Saharan Africa (SSA) (Ayanlade et al., 2022; Conway et al., 2015; Field et al., 2011; Trisos et al., 2022). Consequently, a significant negative impact of climate change on food production is expected globally (Jägermeyr et al., 2021; IPCC, 2022), but particularly in SSA countries, whose economies and livelihoods depend on rainfed agriculture (FAO, 2020; Pickson and Boateng, 2022). Combined with infertile soils and often degraded soils (Baumgartner and Cherlet, 2015; Pasley et al., 2020), crops will suffer concurrent water and nutrient limitations. Among the nutrients, nitrogen (N) is of particular significance and its unavailability constrains plant growth and development in most terrestrial ecosystems (Fageria and Baligar, 2005; Harpole et al., 2011). Nitrogen deficiency is a major contributor to the large African crop yield gap (Falconnier et al., 2020; Gerber et al., 2024; Van Ittersum et al., 2016; Pasley et al., 2020). As water and nutrients are taken up from below ground, water and nutrient limitations primarily need to be overcome in soils. However, below-ground adaptive strategies have received less attention than above-ground strategies. Evident knowledge gaps, specifically relating to processes at the plant-soil interface—the rhizosphere—need to be explored. Among the below-ground traits, root properties, such as root length and rooting depth, play a critical role in the uptake of mobile resources such as water and N (Mommer et al., 2016). However, under drought, the interactions at the root and soil interface are critical in overcoming resource limitations, including N, since water and nutrients traverse the rhizosphere before being taken up by the plant (Abdalla et al., 2022; Cai et al., 2023). Thus, the rhizosphere is considered a critical interface governing resource acquisition by the plant (Vetterlein et al., 2020; Wang et al., 2022). Consequently, identifying effective rhizosphere traits is key in overcoming N scarcity while promoting sustainable agriculture (Hallett et al., 2022).

The interaction with and support of the microorganisms in the rhizosphere (the rhizo-microbiome), is one of the mechanisms by which plants enhance resource acquisition following drought (Jamil et al., 2022; Phour et al., 2020). Particularly, arbuscular mycorrhiza fungi (AMF), obligate symbionts with plant roots, are specialists among the rhizo-microbiome relying fully on their hosts for their C requirements (Chowdhury et al., 2022; Thirkell et al., 2020) in exchange for mineral nutrients (Thirkell et al., 2021; Wipf et al., 2019). Allocation of freshly assimilated C by the plant to symbiotic and freely associated members of the rhizo-microbiome is crucial for fueling the microbial activity and thus for enhancing plant nutrient uptake (Fellbaum et al., 2012; Kelly et al., 2022).

Previous studies have shown that the AMF hyphae-soil interface “the mycosphere” enhances the soil volume exploited for nutrient acquisition beyond the rhizosphere region (Faghihinia et al., 2023; Marschner, 2012; Miyata and Umehara, 2024; Wang et al., 2022). This subsequently improves the availability of nutrients with limited mobility in soils, such as phosphorus (P) (Liang et al., 2022; Qi et al., 2022; Tibbett et al., 2022). While AMF have been shown to enhance N uptake in their symbiotic plants (Thirkell et al., 2016; Shen et al., 2023; Wu et al., 2024; Xue et al., 2024), the mechanisms governing N acquisition, particularly within the rhizosphere, remain unclear and require further investigation. Disrupted connectivity of water films in dry soils reduces the mobility of all nutrient ions irrespective of their speciation (Xue et al., 2017; Bauke et al., 2022; Huntley, 2023). Thus, the role of AMF in bridging air gaps in dry soil presents an opportunity that might be exploited to increase their relevance for N transportation. One way, for example, lies in optimizing their N uptake by extending their hyphal networks into regions inaccessible by plant roots. The fungal hyphae, being much thinner than roots, penetrate small soil pores and maintain functionality in drier environments (Dodd et al., 2000; Zou et al., 2015; Diagne et al., 2020; Abdalla et al., 2023; Hammer et al., 2024). This allows them to access residual water and nutrients, such as N, that remain in the soil micropores despite overall soil drying (Clark and Zeto, 2000; Smith et al., 2010).

Besides their role in nutrient transport, AMF also modify the microbial community in the (rhizo-) hyphosphere (Faghihinia et al., 2023), which may further affect the mobilization of organic N by exoenzymes (Fall et al., 2022; Fellbaum et al., 2012; Jamil et al., 2022). Among the various organic N pools in soil, two key biomolecules, i.e., proteins and chitin, contain the majority of the hydrolyzable N pool, which is quantitatively important for crop N nutrition (Andersson and Berggren, 2005). They are derived from the two main organic matter sources, plant and microbial biomass. Proteins originate from both plants and microorganisms, whereas chitin and peptidoglycan are derived from the cell walls of the soil microbiome. Consequently, protease and chitinase activities play an important role in organic compound degradation and N cycling, especially under reduced N availability (Ndabankulu et al., 2022; Uwituze et al., 2022). Extracellular enzyme activities in soil depend strongly on the soil moisture status since i) water films are the medium in which substrates are transported to the active center of the enzymes or ii) enzymes increasingly adsorb to soil particles under shrinking water films (Geisseler et al., 2011; Tecon and Or, 2017; Schimel, 2018; Guber et al., 2022; Qu et al., 2023). However, it remains to be elucidated if drought-tolerant crops such as sorghum have developed specific strategies to efficiently exploit the soil organic N pool even under water scarcity. If so, it is still an open question to what degree this pool is of use for sorghum N nutrition.

Sorghum [*Sorghum bicolor* (L.) Moench] is one of the major staple food crops, ranked the fifth-most cultivated cereal after wheat, maize, rice, and barley (Ananda et al., 2020; Stefoska-Needham and Tapsell, 2020; Takanashi, 2023). It is the second most important cereal after maize in SSA (Haussmann et al., 2002; FAO, 2023; Yarnell, 2008) where unpredictable drought stress constitutes a major constraint for crop production (Dicko et al., 2006; Hadebe et al., 2017). Although sorghum is considered a drought-tolerant crop, water deficiency still affects its nutrient mobilization and uptake, with negative impacts on yield (Abreha et al., 2022; Ajeigbe et al., 2018; Yu et al., 2015). Previous studies have shown that AMF colonization of sorghum roots leads to enhanced N and P uptake (Deepadevi et al., 2010; Symanczik et al., 2018; Wang et al., 2019). However, an extensive understanding of the influence of water scarcity on AMF and associated enzyme activities is still limited. Thus, this study aims to fill the crucial gaps in understanding how sorghum responds to N limitation under drought.

**Figure 1:**
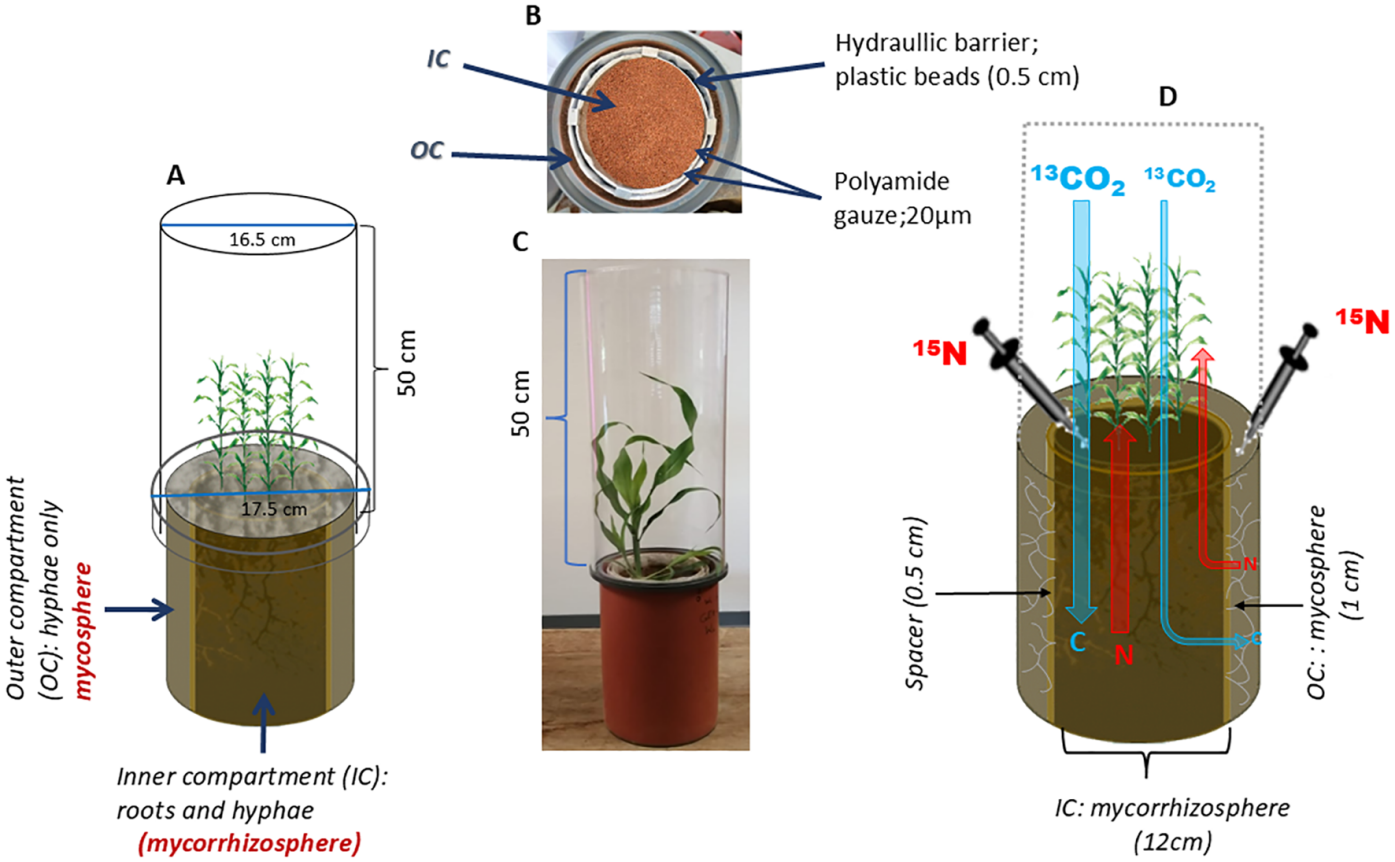
Schematic diagram of **(A)** a double ring pot (DRP) showing the inner and outer compartments. **(B)** Aerial view of the DRP. **(C)** DRP with sorghum plants and the labeling chamber (transparent Plexiglas). **(D)** Isotopic labeling application.

To address these open questions, we designed a double-ring pot (DRP) experiment with two compartments separated by a 0.5 cm “gap” (**Figure 1**). Only AMF hyphae had access to the outer hyphal compartment, the mycosphere, whereas plant-roots and hyphae interacted jointly in the inner compartment of the pot, the mycorrhizosphere. We studied three sorghum genotypes, known to be adapted for cultivation under strong water deficit conditions and thus assumed to be well-adapted to nutrient uptake under drought conditions. We selected a local landrace Makueni local (Mkl), widely cultivated in Kenya; an open-pollinated variety Gadam (Gd), known to be highly drought tolerant; and a commercial hybrid IESH 22012 (IESH) that has been released by the International Crop Research Institute for Semi-Arid Tropics (ICRISAT), as a new drought-tolerant hybrid. The three sorghum genotypes were established at optimum [80% of water holding capacity (WHC)] and drought (30% of WHC) water levels.

This study aimed to 1) determine the effects of drought on N uptake in plant biomass of three sorghum genotypes; 2) assess the relative contribution of AMF to mineral N uptake of sorghum from mycorrhizosphere and mycosphere under optimal and drought conditions; 3) determine whether sorghum C allocation contributes to rhizosphere adaptation strategies that mitigate combined N and water stress; and, 4) investigate the possible shifts in activities of rhizo-(hyphosphere) enzymes degrading soil N polymers in response to drought. We hypothesized that reduced availability of mineral N under drought worsens plant N nutrition (H1) and that drought enhances AMF colonization of sorghum roots as a strategy to cope with water limitation (H2). This may be attributed to AMF hyphae’s ability to bridge air gaps between the disrupted water films inaccessible to roots, improving mineral N acquisition and transportation to the plant roots. We expect a relative increase in C allocation below ground under drought compared to well-watered conditions, which may support the microbiome in its functional contribution to plant nutrition (H3). We further hypothesized a decrease in enzyme activities and a concurrent impact on plant N nutrition under drought compared to optimal conditions (H4).

## 2 Materials and methods

### 2.1 Soil characteristics

The soil was obtained from the top 25 cm of an agricultural Acrisol (IUSS Working Group WRB, 2015) in Kitui (1° 22’ S and 37° 59’ E) located in lower eastern Kenya with a mean temperature of 24°CC and an annual rainfall ranging between 500–700 mm. The soil has a sandy and loamy texture with a total C content of 0.6%, a total N content of 0.07%, a pH (H_2_O) of 5.44, and a WHC of 32%. The soil was air-dried and passed through a 4-mm sieve prior to seed sowing. It is important to clarify that soil was not inoculated with AMF species but, before sampling, we made sure to select soil from a field site where frequent sorghum cultivation took place and where we expected a sufficient spore load in the soil for effective mycorrhization.

### 2.2 Experimental design

Double-ring pots (**Figure 1**) with a height of 30 cm were designed to separate the inner compartment (IC)—the mycorrhizosphere (12 cm diameter)—from the outer compartment (OC)—the mycosphere (1 cm width). The IC was separated from the OC by a 0.5 cm gap filled with hydrophobic plastic (2–3 mm) beads acting as a hydraulic barrier. Two layers of 20 µm polyamide gauze at the sides of the hydraulic barrier ensured that only hyphae and no fine roots could reach the OC. Sorghum (*Sorghum bicolor* MOENCH) seeds, obtained from ICRISAT, Kenya, were sterilized in a 10% hydrogen peroxide solution for 10 minutes (Gaume et al., 2001; Davoudpour et al., 2020). Three sorghum genotypes, i.e., Mkl, Gd, and IESH, representing different levels of breeding effort, were used. The genotypes (Mkl, Gd, and IESH) have varying levels of extensive selection to enhance their drought tolerance, with the landrace.

(Mkl) having the least, followed by the open-pollinated variety (Gd), while the hybrid (IESH) had the highest selection intensity. Five seeds of each genotype were sown in the IC of the double-ring pot and were thinned to one single plant when the third leaf (after 15 days) appeared.

At the beginning of the experiment, a half dose of the recommended seed starter fertilization in solid form of 135 mg NPK [0.1285 g KCl, 0.0251 g ammonium phosphate (NH_4_)(H_2_PO_4_), and 0.08 g urea CH_4_N_2_O] was mixed with the soil when filling each pot. This enabled germination and initial growth but then led to a transition towards nutrient deficiency, allowing the observation of the plant’s response to combined nutrient and drought stress. The fertilization was applied relative to the proportion of soil mass in the outer and inner compartments (3:1), so that the content of the applied fertilizer in mg/g soil was identical in both compartments. A total of 48 double-ring pots were prepared for a full factorial design with four replicates and two water levels: optimum (80% of WHC) and drought (30% of WHC). With 30% WHC, the drought-sensitive sorghum cultivars will fail to produce a yield, whereas the drought-tolerant ones may still produce a yield. This level was chosen as adaptive traits may have the highest impact in this range of soil moisture (Sauer et al., 2024; Loftus et al., 2025).

For the first week of growth, the WHC of all pots was maintained at 80%. We weighed each pot every second day and manually watered with deionized water the outer or inner compartments to ensure consistent moisture levels for optimal plant establishment. Plant-free control pots were also weighed to quantify the evaporation of water per gram of soil. This was assumed to equal the evaporation per gram of soil of the outer ring, which was then used to calculate the amount of water to be added to the outer ring to compensate for evaporation. The difference between the total weight loss and the amount of water added to the outer ring was, in consequence, assumed to be caused by evapotranspiration from the inner ring. This amount was then added, as described above, to the outer ring. After three weeks, 24 pots were no longer watered. The dry-down process was controlled by weighing and recording the soil moisture content (**Supplementary Table S1**) until it reached 30% WHC (drought treatment). The other half of the pots were maintained at optimal conditions (well-watered treatment). The plants were grown in a walk-in growth chamber (Department of Crop Science, University of Göttingen, Germany) with a photoperiod of 12-hr day and night at temperatures of 27°C and 17°C, respectively, and a light intensity of 500 µmol m−² s−¹ from LED fluorescent lamps.

### 2.3 ^15^N tracer application

To trace N acquisition from the IC relative to the OC, ^15^N isotopes (^15^NH_4_)_2_SO_4_ and ^15^N_2_-urea (99 at%, Sigma Aldrich, Munich, Germany) were applied. To minimize the nitrification that may occur due to high initial ammonium concentration, both ammonium and urea were applied together. This allows for a continued supply of ammonium ions through the slow mineralization of urea. Thus, 5 weeks after sowing, approximate concentrations of 1.67 mg ml^-1^ (^15^NH_4_)_2_SO_4_ and 0.85 mg ml^-1 15^N_2_-urea, along with the second half of the starter NPK fertilization in a single pulse, were applied. The tracer solution was applied in the form of eight single injections (eVol displacement pipette, SGE, Australia) of 0.5 ml each either into the IC or the OC, in a quadrilateral pattern to promote a homogeneous distribution of the tracer in the respective compartments.

### 2.4 ^13^CO_2_ pulse labeling

At week 7, single-pot ^13^CO_2_ pulse labeling was carried out to quantify the below-ground C allocation of the individual plants (Kuzyakov and Domanski, 2000; Sommer et al., 2017; Stock et al., 2021). Silicone rubber (Henkel Teroson GmbH, Heidelberg, Germany) of about 5 mm was evenly and seamlessly applied on the soil surface a day prior to the labeling to allow sufficient time to dry and form a tight seal. This prevented the diffusion of the labeled CO_2_ into the soil, thereby avoiding hetero- or autotrophic microbial C fixation leading to an overestimation of the below-ground C respiration. Bespoke transparent polymethyl methacrylate domes with a height of approx. 50 cm and a volume of 10.7 l were placed on each pot and sealed to ensure an airtight system (**Figure 1C**). Before labeling, CO_2_ concentrations in the chambers were lowered by cycling the air through a sodium hydroxide trap (1 M) for 0.5 h. During labeling, the plants were exposed to ^13^C-enriched CO_2_ for 2 h produced by the reaction of 0.0045 g of Na_2_^13^CO_3_ (99 at% ^13^C, Sigma Aldrich, Munich, Germany) with 1 M H_3_PO_4_ added dropwise per pulse, with two pulses applied over a period of 4 h. To ensure uniform circulation of ^13^CO_2_ during labeling, a battery-powered fan was operated inside the chamber. The chamber was opened before and after labeling, and unassimilated ^13^CO_2_ was captured by cycling the air for 0.5 h through 20 mL of a 1 M NaOH solution.

### 2.5 Harvesting and soil sampling

Two days after the ^13^C pulse labeling, the plants and soils were destructively sampled. Shoot and root biomass were separated. The roots were manually removed from the soil and washed with deionized water by sonification to remove the soil adhering to the roots. After this, the samples were freeze-dried and weighed to obtain dry weights. Soil samples were taken from each compartment, homogenized, and stored at 4°C prior to subsequent enzyme assay and microbial biomass ^13^C analysis.

### 2.6 Analysis of ^13^C and ^15^N in soil and plant biomass

To determine C and N content and their isotopic composition (δ^13^C and δ^15^N), the dry biomass of plant and soil samples were milled with MM2000 (Retsch, Haan, Germany), and a subsample of approximately 2 mg, 1.5 mg, and 30 mg of root, shoot and soil, was weighed in tin capsules, respectively. The C and N contents were determined by an elemental analyzer (Flash 2000, ThermoFisher Scientific, Bremen, Germany) coupled via a Conflow to an isotope ratio mass spectrometer (IRMS) (Delta V Plus, ThermoFisher Scientific, Bremen, Germany). Measurements were conducted at the Centre for Stable Isotope Research and Analysis (KOSI); University of Göttingen. To calculate the ^15^N recovery in plant material, the atom% ^15^N of sorghum plants not supplied with ^15^N served as a natural abundance control. The ^15^N recovery was calculated by Equation 1, as follows:


(1) $${[}^{15}\text{N}]{\;}_{\text{acqui}}={\left[\text{N}\right]}_{\text{lab}}\times \frac{\left({\text{at\%}}^{15}{\text{N}}_{\text{lab}}-{\text{at\% }}^{15}{\text{N}}_{\text{nat}}\right)}{{\text{(at\% }}^{15}{\text{N}}_{\text{tracer }}-{\text{at\% }}^{15}{\text{N}}_{\text{nat}})}$$

where [^15^N] _acqui_ is the ^15^N amount taken up [^15^N in plant dry biomass (mg plant^-1^)], [N]_lab_ is N amount of the labeled plant material [N in total plant dry biomass (mg plant^-1^)], at% ^15^N_lab_ is the at% ^15^N of the labeled plant material, and at%^15^N_nat_ and at% ^15^N_tracer_ are the unlabeled plant material and the injected tracer (99 at% ^15^N), respectively. The N amount [N]_lab_ was derived by multiplying the weight of shoot or root biomass with the biomass N concentration [mg g^-1^] of the respective plant compartment.

The ^15^N recovery in the percentage of applied tracer per plant (derived from the inner or outer ring of the pot, respectively) was calculated by dividing [^15^N] _acqui_ of the shoots and roots (in mg) by the ^15^N amount (mg) applied either to the inner or outer compartment and multiplied by 100.

### 2.7 Microbial biomass ^13^C determination

The soil microbial biomass C (MBC) was extracted using the chloroform-fumigation extraction method (Potthoff et al., 2003; Wu et al., 1990). Briefly, two samples of 10 g fresh soil, stored at 4°C (for maximally 7 days), were taken; one sample was first fumigated in a chloroform atmosphere for 24 h in a desiccator to lyse the microbial cells membranes, while the other sample was left unfumigated. Water-dissolvable C was extracted from both samples by shaking them in 40 ml 0.05 M K_2_SO_4_ for 1.5 h. The samples were centrifuged at 2,000 rpm for 10 minutes, the supernatant filtered, and the solutions stored at -20°C until analysis on a N/C 2100 element analyzer (Jena Analytik, Jena, Germany). Afterward, the extracts were lyophilized and approximately 25 mg (fumigated) and 35 mg (unfumigated) freeze-dried powder were weighed into tin capsules and measured as described above (Section 2.6).

The ^13^C_acqui_fum/unfum_ was calculated according to the mixing model, which is shown in Equation 1 for N and was modified for C as shown in Equation 2. We first calculated the ^13^C_aqui_fum_ and ^13^C_acqui_unfum_ as separate independent pools, using the ^13^C values of labeled and unlabeled microbial biomass extracts, respectively, and using the enrichment of 99 at% for the ^13^CO_2_ tracer, as added to the chamber.


(2) $${[}^{13}\text{C}]{\;}_{\text{acqui}\_\text{fum/unfum}}={\left[\text{C}\right]}_{\text{lab}(\text{fum}/\text{unfum})}\times \frac{\left(\text{at}{\%}^{13}{\text{C}}_{\text{lab}}-\text{at}\%{\;}^{13}{\text{C}}_{\text{nat}}\right)}{\text{(at}\%{\;}^{13}{\text{C}}_{\text{tracer }}-\text{at}\%{\;}^{13}{\text{C}}_{\text{nat}})}$$

where [^13^C]_acqui_ is the ^13^C amount allocated below ground into the extractable pool with and without fumigation (µg pot^-1^), [C]_lab(fum/unfum)_ is the amount of C extracted from the labeled fumigated and unfumigated samples and scaled per pot (µg pot^-1^) at% ^13^C_lab_, and at%^13^C_nat_ is the at% ^13^C value of the extract from the labeled and unlabeled pots, respectively, while at% ^13^C_tracer_ is the atom% value of the injected tracer (here 99 at% ^13^C), respectively.

Microbial biomass C content per µg of extracted soil was multiplied by the amount of soil in the respective ring to calculate the C pool [C]_lab_ of the fumigated and unfumigated extracts from the soil of the respective rings. The ^13^C-MBC was calculated as the difference between ^13^C in fumigated and non-fumigated soil extracts and divided by a factor of 0.45 to correctly account for uncomplete cell lysis extraction efficiency (Wu et al., 1990), as shown in Equation 3.


(3) $${\;}^{13}\text{C}-\text{MBC}={(}^{13}{\text{C}}_{\text{acqui}\_\text{fum}}-{\;}^{13}{\text{C}}_{\text{acqui}\_\text{unfum}})/0.45$$

where ^13^C acqui_fum and unfum indicate acquired ^13^C from fumigated and non-fumigated soil extracts, respectively.

### 2.8 AMF colonization evaluation

After harvest, fresh roots were collected, washed, and weighed. A subsample of approximately 10% of root mass was taken to estimate AMF root colonization. Roots were stained according to the slightly modified method of Vierheilig et al. (1998) where the roots were subjected to a bleaching step by heating them in 5% KOH in a water bath at 95°C for 4 minutes (if needed, 4 minutes of bleaching were added). Thereafter, the roots were washed with tap water for 3 to 5 minutes and the water was changed once. All the roots were stained using 5% ink (Pelikan ink 4001^®^, Pelikan, Hannover) in an acetic acid solution for 5 minutes at a temperature of 95°C. Following this, the roots were de-stained in acidified water for 30 min. Roots were kept in 80% glycerol at 4°C until microscopic analysis (Stock et al., 2021). AMF colonization was quantified by counting vesicles, arbuscules, and intraradical hyphae under a light microscope (Olympus BX40 with Olympus CMOS-camera SC50M, 4.9 Mpx resolution) at a magnification of x150 (Olympus Ach x10/0.25 ph ∞ 0.17, ocular x15, Olympus Europe SE & Co. KG, Hamburg, Germany). Ten root segments, each of one cm length, were analyzed for each plant genotype. Grid squares were then classified and counted as either containing only root tissue (–) or containing root tissue and fungal structures (+). The percentage of roots colonized by AMF was calculated by the relation of squares containing root and fungi structures jointly to the total number of squares containing root segments with or without fungi. The results were expressed as a percentage of the root area colonized.

### 2.9 Enzyme assays

To determine the potential activities of C- and N-cycling exoenzymes, we measured leucine aminopeptidase (LAP) and poly-N-acetyl-glucosaminidase (chitinase) activities. The LAP cleaves the N-terminal from proteins and peptides (Greenfield et al., 2021; Matsui et al., 2006); while chitinases hydrolyze chitin to low molecular weight chitooligomers (Hoang et al., 2016; Zhang et al., 2020). Both are significant N- and C-acquiring enzymes and broadly representative of C/N cycling activities (Cenini et al., 2016). Fluorogenically labeled substrates (all from Sigma Aldrich, Germany), 4-MUF-N-acetyl-b-D-glucosaminide (MUF-NAG) and L-Leucine-7-amino-4-methylcomarin hydrochloride (AMC-LAP), were used to assess the enzyme activities for chitinase and LAP respectively (German et al., 2011; Marx et al., 2001). The enzymes’ activities were determined over a range of substrate concentrations from low to high (0, 5, 10, 15, 20, 25, and 75 μmol g^-1^ soil) at room temperature. Approximately 1 g of fresh soil was mixed with 50 ml of sterile water and placed on a horizontal shaker for 30 minutes. After low-energy sonification for 2 minutes, the 50 μL soil solution and 50 μL MES and Trizma buffer for chitinase and LAP, respectively, were pipetted into 96-well black microtiter plates. Finally, 100 μL of each substrate in a series of 0, 5, 10, 15, 20, 25, and 75 μmol g^-1^ soil were added to the wells. The fluorescence was repeatedly measured at 0, 1 hour, and 2 hours after adding the soil suspension, substrate, and buffer at an excitation wavelength of 360 nm and an emission wavelength of 450 nm, on a fluorescent microplate reader (Victor^3^ 1420-050 multi label counter PerkinElmer, USA) (Razavi et al., 2015). Enzyme activities were expressed as MUF/AMC release in nmol g^-1^ soil h^-1^. The assays of each enzyme at each substrate concentration were performed in three analytical replicates. The Michaelis–Menten equation (Equation 4) was used to calculate the parameters of enzyme activities V_max_ and K_m_.


(4) $$\text{V}=\frac{{\text{V}}_{\text{max }}\times [\text{S}]}{{\text{K}}_{\text{m }}+[\text{S}]}$$

where V_max_ is the maximal rate of enzymatic activity, Km is the half-substrate concentration at the half-maximal rate and S is the substrate concentration.

### 2.10 Statistical analyses

Statistical analyses were performed using statistical software R, version 4.2.0. (R Core Team, 2022). Prior to running analyses, data were checked for normality and homogeneity of variance using Shapiro–Wilk and Levene’s test, respectively. The data violating normal distribution were first logarithmically transformed before further analysis. Data with eight replicates were tested by two-way ANOVA, where the water content and genotypes were used as predictor variables for the plant biomass, ^13^C allocation, and enzyme data analyses, while three-way with only four replicates ANOVA was performed on ^15^N recovery with root presence as the third predictor. Subsequently, Tukey’s HSD *post hoc* test was performed at a significance level of α = 0.05 to separate the means. Simple linear regression analysis was conducted to assess the relationship between N acquisition and enzyme activity, assuming linearity, normal distribution of residuals, and homoscedasticity.

## 3 Results

### 3.1 Plant biomass, N accumulation, and mycorrhizal colonization

**Figure 2:**
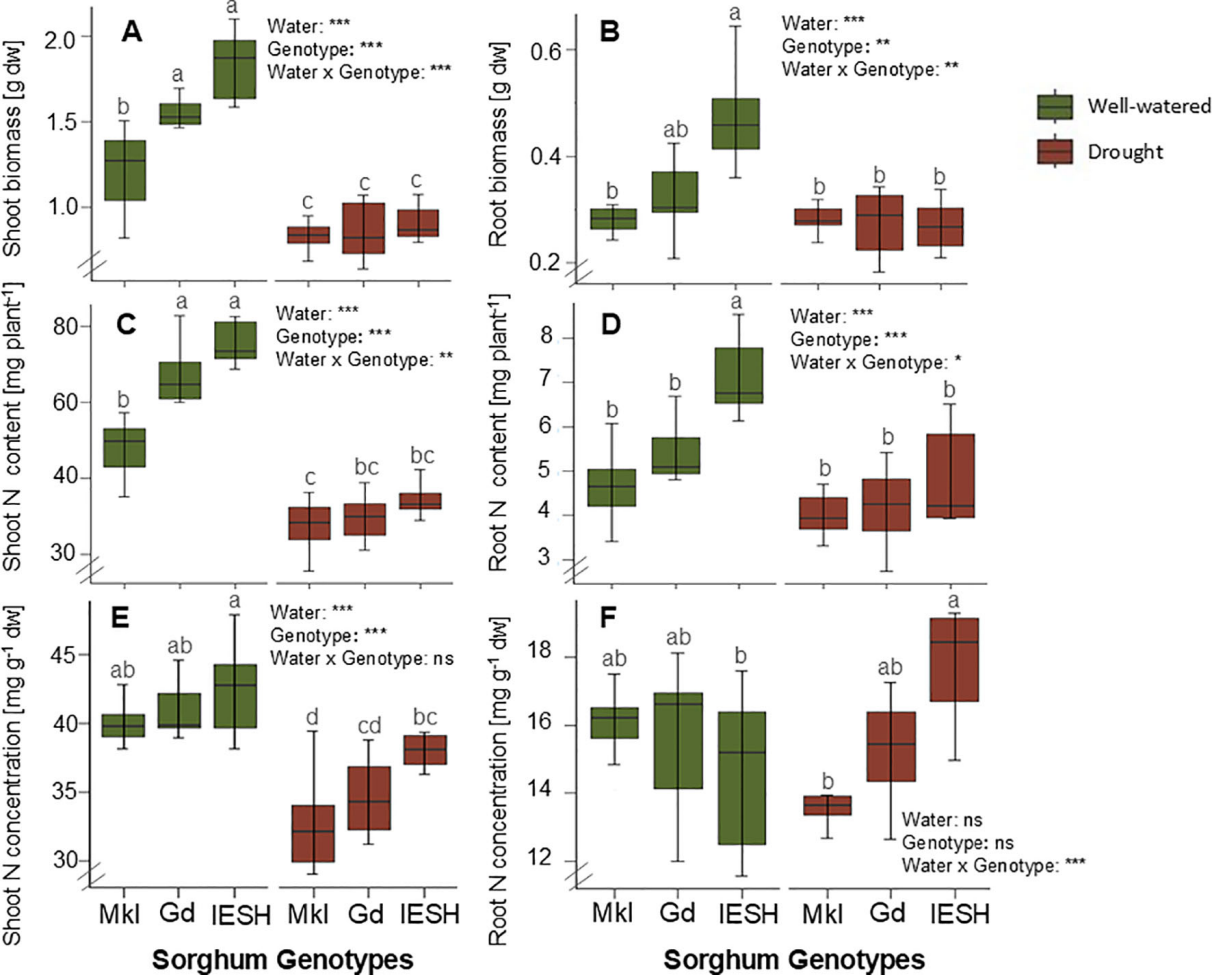
**(A)** Shoot and **(B)** root biomass, **(C)** shoot and **(D)** root total nitrogen (TN) content, and **(E)** N concentration per g dry weight in shoot and **(F)** root of sorghum genotypes under well-watered (green) and drought (brown) conditions. Data are presented as means ± SE (n=8). Different letters indicate significant differences (p<0.05). Sorghum genotypes: Mkl, Makueni local; Gd, Gadam; IESH, IESH 22012. P-values of ANOVA of water availability, genotypes, and their interactions are indicated as ***p<0.001, **p<0.01, and *p<0.05, and ns indicates not significant.

Drought substantially decreased (p<0.0001) plant biomass compared to growth under optimal water conditions (**Figures 2A, B**). Shoot dry weights of the three sorghum genotypes were considerably reduced under drought stress (**Figure 2A**), with IESH showing the highest reduction of 50.7%, followed by Gd and Mkl with 44% and 30%, respectively. Similarly, the dry weight of the roots decreased under drought but was significant only for IESH with a 46% decrease (**Figure 2B**). Under drought, the root:shoot ratio significantly increased for Mkl while that of Gd and IESH remained relatively unaffected (**Table 1**).

**Table 1:** Root/shoot ratio and percentage of AMF root colonization in sorghum genotypes under well-watered and drought conditions.

Water availability	Genotype	Root/shoot ratio	AMF hyphae [% of colonized root area]
Well-watered	Mkl	0.24b	28.2± 3.26abc
	Gd	0.21ab	42.82 ± 4.96a
	IESH	0.28ab	43.01 ± 4.98a
Drought	Mkl	0.35a	18.97 ± 2.01c
	Gd	0.33ab	32.03 ± 3.71ab
	IESH	0.3ab	25.07 ± 2.90bc
Statistical significance ANOVA			
Water		**	***
Genotype		ns	**
Water x genotype		ns	ns

Data are presented as means ± SE (n=8). Different letters indicate significant differences (*p*<0.05). Sorghum genotypes: Mkl, Makueni local; Gd, Gadam; IESH, IESH 22012. P-values of ANOVA of water availability, genotypes, and their interactions are indicated as ***p<0.001, **p<0.01, and *p<0.05, and ns indicates not significant.

Drought decreased (p<0.0001) total nitrogen (TN) content by 13%–17.5% (**Figure 2C**) and N concentration per dry weight in shoot biomass (**Figure 2E**). However, only IESH showed a significant reduction of its N content with a water deficit in root tissue, while that of Gd and Mkl remained unchanged (**Figure 2D**). Biomass and N content among the studied genotypes did not vary under drought conditions (**Figures 2A–D**). All individuals were colonized by AMF. Water availability significantly influenced (p<0.0001) the degree of colonization (% of colonized root area) (**Table 1**, ANOVA). Drought conditions significantly decreased root colonization for IESH, while Gd and Mkl were not significantly affected (**Table 1**). The genotype significantly influenced (p<0.001) AMF colonization. Mkl roots showed the lowest colonization of all genotypes irrespective of soil moisture levels (**Table 1**).

### 3.2 ^15^N recovery

Water availability, genotype, and root presence/absence in the compartment of the ^15^N application, and their interactions significantly affected ^15^N recovery in both above- and below-ground biomass (**Table 2**). All genotypes displayed significantly higher recoveries of the mineral ^15^N applied in the mycorrhizosphere compared to that from the mycosphere. For example, the uptake of ^15^N from the mycorrhizosphere into the shoots was 7–17 times higher than the uptake from the mycosphere. In roots, ^15^N uptake from the mycorrhizosphere was 11–33 times higher than uptake from the mycosphere.

**Table 2:** Effects of water availability, genotype, soil compartment (where the tracer was applied), and their interactions on 15N uptake into shoot and root biomass of the sorghum genotypes as analyzed by three-way ANOVA.

	Statistic	Water availability	Genotype	Soil compartment	Water level x genotype	Water level x soil compartment	Genotype x soil compartment	Water level x genotype x soil compartment
**Shoot**	F	7.6412	7.31	389.47	1.28	63.82	4.26	1.10
	P	<0.009**	<0.002**	<0.0001***	ns	<0.0001***	<0.02*	ns
**Root**	F	52.62	7.79	639.37	1.147	157.156	7.41	1.45
	P	<0.0001***	<0.002**	<0.0001***	ns	<0.0001***	<0.002**	ns

Data are presented as means ± SE (n=4). Different letters indicate significant difference (p<0.05). Sorghum genotypes; Mkl - Makueni local, Gd - Gadam and IESH - IESH 22012. p-values of ANOVA of water availability, genotypes, soil compartment and their interactions are indicated as ***p<0.001, **p<0.01, *p<0.05 and ns - not significant.

In contrast, a significantly higher contribution of the mycosphere to plant ^15^N recovery was observed under drought conditions for all genotypes. While under well-watered conditions only approximately 1% of the ^15^N uptake resulted from the outer mycosphere relative to the inner mycorrhizosphere compartment, the contribution of the mycosphere compartment increased to 9% –44% of the ^15^N uptake under drought. The highest recovery was obtained by the genotype Gd.

**Figure 3:**
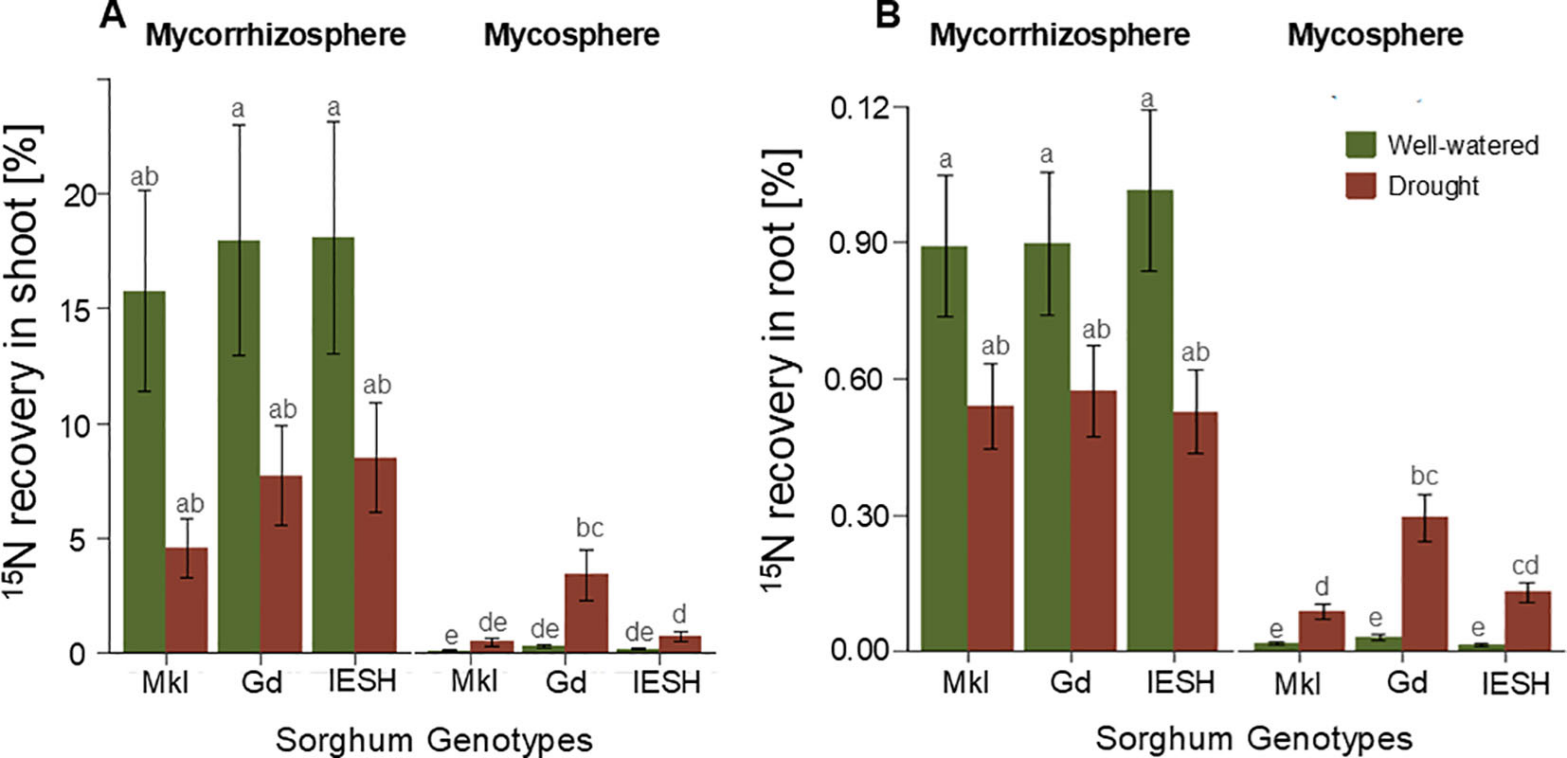
^15^N recovery of applied tracer N in **(A)** shoot and **(B)** root of sorghum genotypes under well-watered (green) and drought (brown) conditions when labelled in the mycorrhizosphere (left) and mycosphere (right). Data are presented as means ± SE (n=4). Different letters indicate significant difference (p<0.05). Sorghum genotypes; Mkl-Makueni local, Gd-Gadam and IESH 22012.

In shoots, Mkl and IESH showed a 4-fold and Gd a12-fold higher ^15^N recovery from the outer mycosphere compartment under drought than under well-watered conditions. In the roots, Mkl had a 5-fold, IESH had a 9-fold, and Gd had a 10-fold higher ^15^N recovery from the outer mycosphere compartment under drought compared to well-watered conditions, respectively (**Figures 3A, B**, for at% data, see **Supplementary Table S2**). The sorghum genotypes showed a significant variation in their ^15^N recovery from the mycosphere in both shoot and root biomass (**Figures 3A, B**). Gd showed higher recovery than Mkl, while IESH was not significantly different from either of the other two genotypes.

### 3.3 Below-ground plant C allocation into microbial biomass

**Figure 4:**
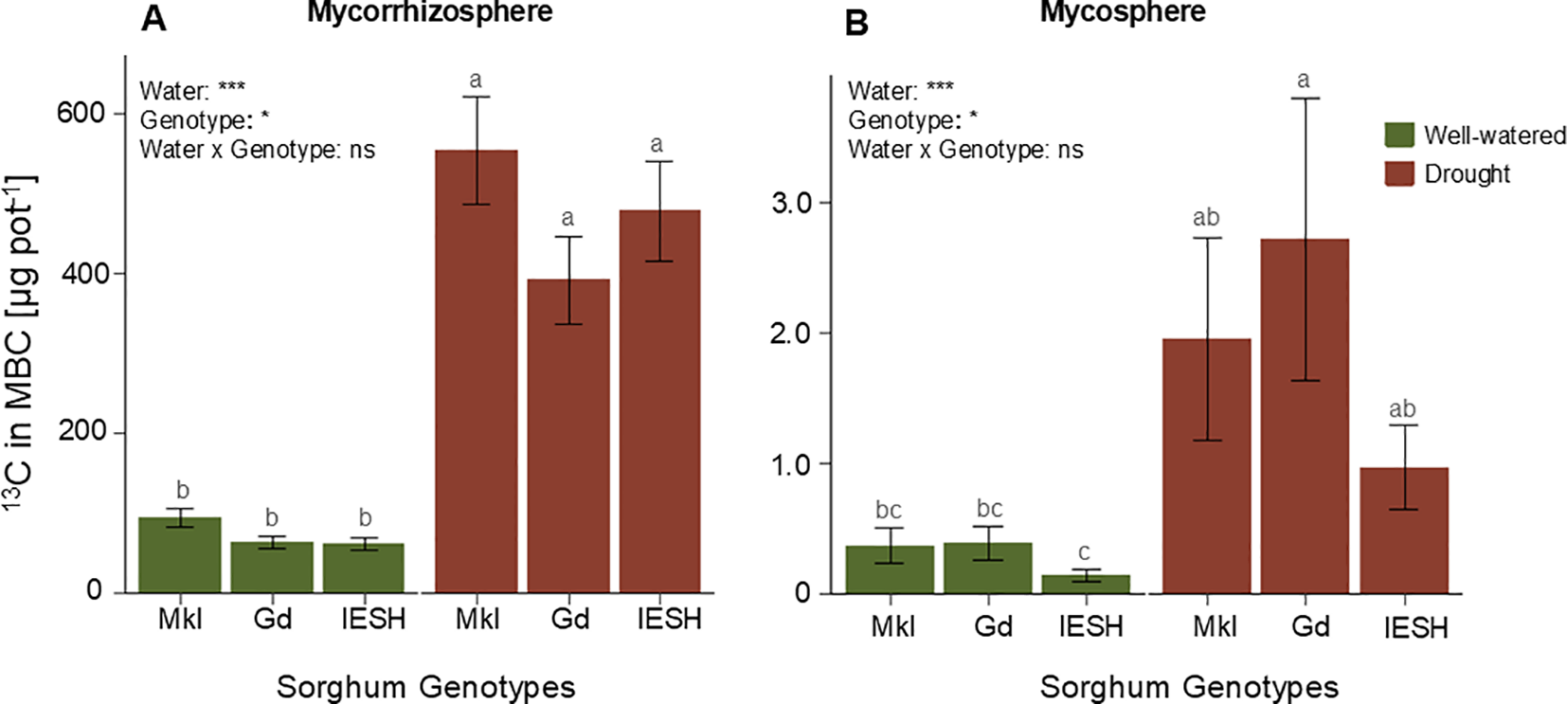
^13^C allocation into the microbial biomass pool **(A)** in the mycorrhizosphere and **(B)** in the mycosphere of the three sorghum genotypes under well-watered (green) and drought (brown) conditions. Data are presented as means ± SE (n=8). Different letters indicate significant differences (p<0.05). Sorghum genotypes: Mkl, Makueni local; Gd, Gadam; IESH, IESH 22012. P-values of ANOVA of water availability, genotypes, and their interactions are indicated as ***p<0.001, **p<0.01, and *p<0.05, and ns indicates not significant.

Drought stress resulted in a significant increase (p<0.0001) in the allocation of recently assimilated ^13^C into the microbial biomass pool, both in the mycorrhizosphere and mycosphere compared to the well-watered pots with no difference among the three genotypes (**Figures 4A, B** **).** The amount of ^13^C incorporated in microbial biomass under drought was 5- to 7-fold higher than in the optimal moisture treatment in the mycorrhizosphere (**Figure 4A**). Although there was a lower recovery of recently assimilated C in the microbial biomass of the mycosphere compared to mycorrhizosphere by a factor of 50, drought stress also boosted the incorporation of freshly assimilated C in this mycosphere compartment, reaching increases of 2.5 to 6-fold (**Figure 4B**).

### 3.5 Enzyme kinetic parameters

**Figure 5:**
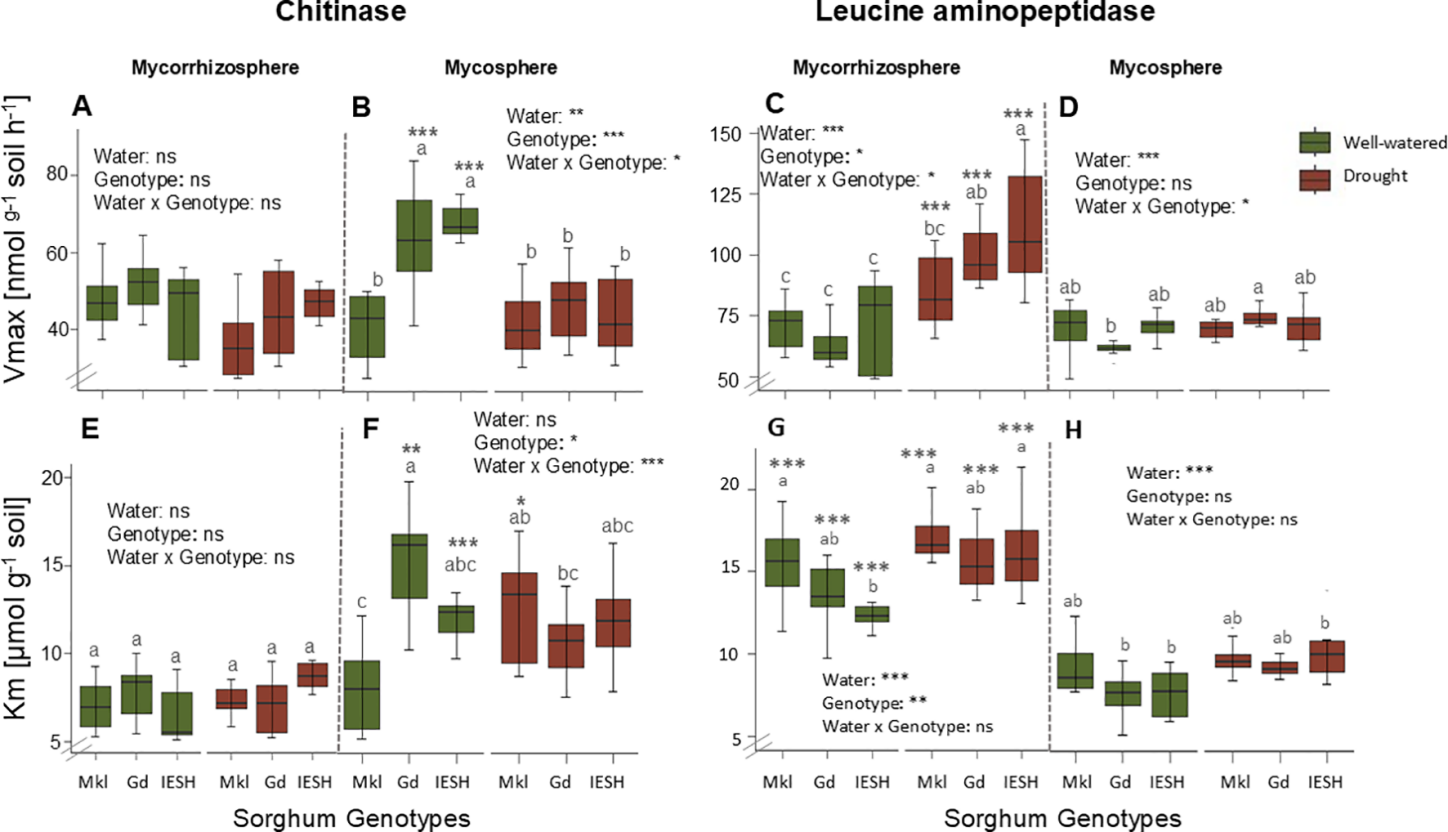
**(A, B)** Chitinase and **(C, D)** leucine aminopeptidase maximal activity (Vmax, nmol g^-1^ soil h^-1^). **(E, F)** Chitinase and **(G, H)** leucine aminopeptidase Michaelis–Menten constants (Km, μmol g^-1^ soil) of the mycorrhizosphere and mycosphere soil for the three sorghum genotypes under optimal water (green) supply and drought (brown) conditions. Data are presented as means ± SE (n=8). Asterisks indicate significant differences between the compartments, i.e., the mycorrhizosphere and mycosphere. Sorghum genotypes: Mkl, Makueni local; Gd, Gadam; IESH, IESH 22012. P-values of ANOVA of water availability, genotypes, and their interactions are indicated as, ***p<0.001, **p<0.01, and *p<0.05, and ns indicates not significant.

Water content, genotypes, and their interactions had no effect on maximal potential enzyme activities (V_max_) of chitinase in the mycorrhizosphere (**Figure 5A**). Chitinase V_max_ was significantly higher (p<0.001) under optimal compared to drought conditions in the mycosphere of Gd and IESH (**Figure 5B**). However, in the mycorrhizosphere, the maximal chitinase activity of the genotypes did not differ depending on water availability (**Figure 5A**). Root presence resulted in higher (p<0.0001) soil LAP V_max_ values of all the genotypes under drought (**Figure 5C**). However, there was no water effect on the maximal enzyme activity for LAP in the mycosphere apart from for Gd (**Figure 5D**). Water availability, genotype, and their interactions indicated no effect on substrate affinity (K_m_ values) of chitinase in the mycorrhizosphere (**Figure 5E**). However, in the hyphal compartment, drought reduced and raised the chitinase K_m_ values for Gd and Mkl, respectively, while that of IESH remained unaffected (**Figure 5F**). For IESH, drought significantly lowered the affinity of LAP in both compartments (**Figures 5G, H**). Mycosphere K_m_ values of LAP were significantly lower compared to that of the mycorrhizosphere (**Figures 5G, H**).

### 3.6 Relation between N acquisition and enzyme activity

**Figure 6:**
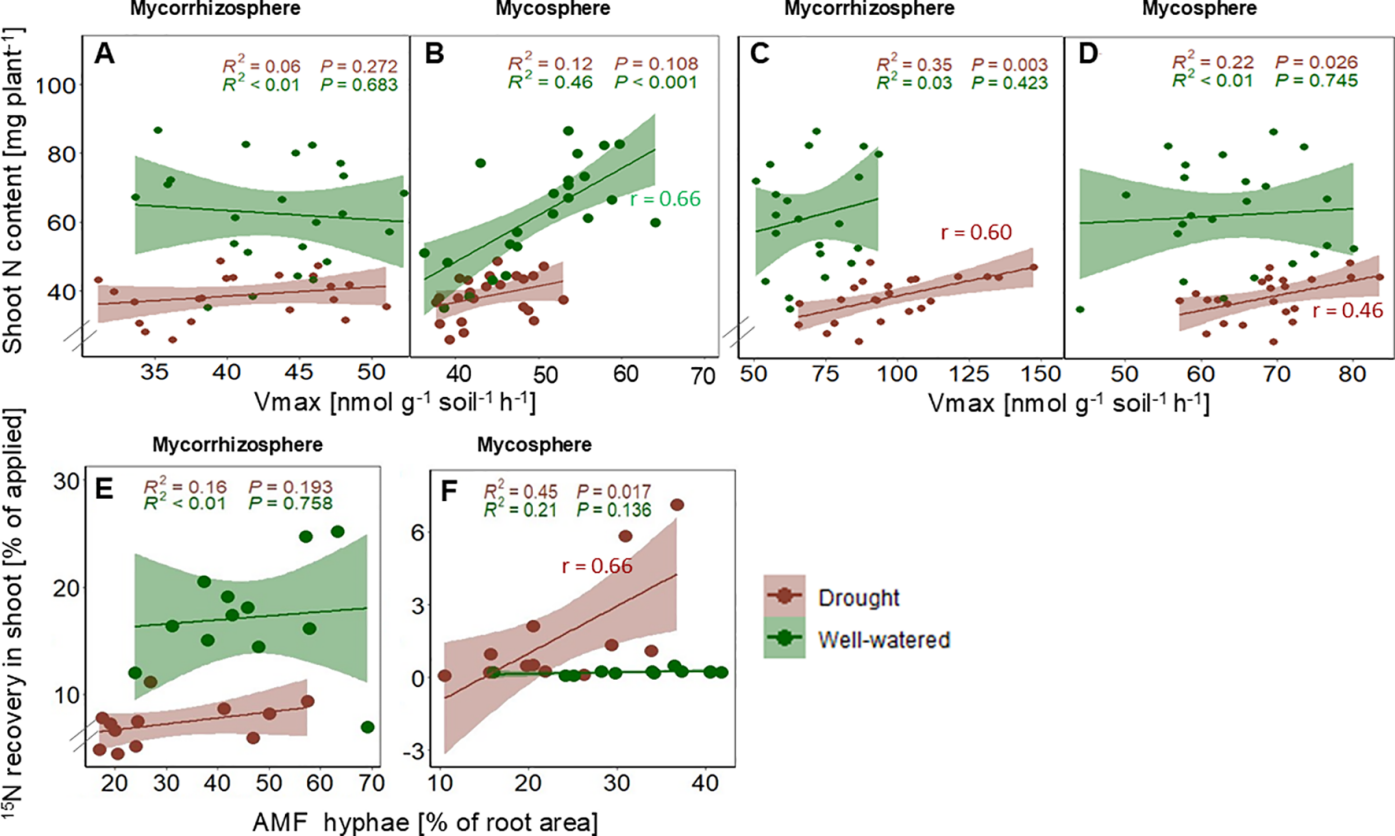
Correlations between shoot N content and chitinase **(A, B)** and leucine aminopeptidase **(C, D)** enzyme activity (Vmax) in the mycorrhizosphere and mycosphere, and between the 15N recovery in shoots (%) and the AMF hyphae colonization **(E, F)** under optimal (green) and drought (brown) conditions. P-values, R^2^ coefficients of determination, and correlation coefficients (r) are indicated.

Shoot N content showed a positive correlation with chitinase enzyme activity (V_max_) (r _(24)_ =0.67, p<0.001) (**Figure 6B**) under optimal water conditions in the mycosphere soil but not in the mycorrhizosphere soil (**Figure 6A**). In contrast, under drought, shoot N content was positively correlated with LAP activity in the mycorrhizosphere (r = 0.60, p<0.01) and the mycosphere (r = 0.46, p<0.05) (**Figures 6C, D**). ^15^N recovery in shoot biomass from mycosphere soil under drought was positively correlated with the degree of root AMF colonization (r =0.66), p<0.05) (**Figure 6F**), whereas there was no correlation observed under optimal conditions (**Figure 6E**).

## 4 Discussion

### 4.1 Drought impact on plant growth, plant N nutrition, and enzymatic N transformations

Drought significantly affects various processes of plant growth (Goche et al., 2020; Seleiman et al., 2021), e.g., it reduced both shoot and root biomass and decreased TN content and N concentration per dry weight in shoot biomass (**Figures 2A–E**). To reduce the loss of water, plants respond to drought through stomatal closure, a primary factor for reduced photosynthesis (Abdalla et al., 2022; Agurla et al., 2018; Buckley, 2019), resulting in a decrease of plant biomass (Hosseini et al., 2022; Khalil et al., 2020; Sigua et al., 2018). Although drought generally reduced sorghum biomass formation in all the genotypes (**Figures 2A, B**), the increased root/shoot ratio (**Table 1**) revealed resource investment in root growth. Root biomass accumulation was particularly enhanced in Mkl and partly in Gd in response to the water deficit (**Figure 2B**). Moreover, the relative increase or absence of change in N allocation in root tissue (**Figure 2D, F**), apart from that in IESH under drought (**Figure 2D**), is an often-observed adaptation strategy of plants to maintain root activity and enhance water and nutrient acquisition from soils under resource-limited conditions (Eziz et al., 2017; Wilschut and van Kleunen, 2021).

Soil moisture availability significantly supports the nutrient uptake of plants (Bista et al., 2018; Liu et al., 2023). Drought not only reduced sorghum biomass but also reduced overall N content in the plant tissue (**Figures 2C, D**), confirming our first hypothesis (H1). One underlying reason may be that the mobile nutrients, including mineral N, are co-transported with the water mass flow to the roots. It is likely that restricted mobility of N ions occurs if the water mass flow is reduced by drought (Araus et al., 2020; He and Dijkstra, 2014). Our findings are consistent with other studies showing that soil moisture deficit conditions suppress root growth, as in IESH genotype (Asif et al., 2020; Kou et al., 2022). Additionally, reduced N content in plant tissues could be due to the loss of contact of the root with surrounding soil particles. Root and root hairs shrink under drought (Duddek et al., 2022), impeding water and nutrient accessibility (Cai et al., 2023; Saengwilai et al., 2021).

Moreover, we observed a reduced V_max_ of chitinase under drought (**Figure 5A**). Soil drying alters the functioning of the microbes and their enzymes (Ahmed et al., 2018) by, e.g., confining substrates in thin water films (Goebel et al., 2011), leading to their sorption to the mineral phase (Singh et al., 2021). Drying also induces enzyme denaturation due to high ion strength or strong interactions in thin water films and reduced enzyme diffusion (Davidson and Janssens, 2006; Guber et al., 2018) This is of special relevance in the iron mineral-rich Acrisol used in this experiment, which has a very high sorption capacity (Kaiser and Guggenberger, 2007; Zhou et al., 2023).

In agreement with our fourth hypothesis (H4), chitinase activity decreased under drought. In contrast, moisture limitation induced an increase of the V_max_ of LAP in comparison to optimal conditions (**Figure 5B**)—a phenomenon already observed in previous studies on LAP (Sanaullah et al., 2011; Zhang et al., 2021). We observed its potential activities to be more pronounced in the mycorrhizosphere than in the mycosphere (**Figure 5B**). Such high maximal LAP activities under drought in root proximity may have been either aided by root priming of the rhizosphere microbiome (Shahzad et al., 2015) or may have been derived from the direct release of LAP from roots (Greenfield et al., 2020, 2021). Reduced tissue N content may have enhanced LAP production to counteract a potential N deficiency, as confirmed by a significant correlation of shoot N content with LAP enzyme activity under drought (**Figures 6C, D**).

The additional release of enzymes upon root and microbial cell death and lysis under drought may perhaps further result in higher enzyme activities of LAP (Turner et al., 2003). Drought consistently lowered the affinity of LAP for its substrates while the response of substrate affinity of chitinase to water scarcity was genotype-specific (**Figures 5G, H**). The change in K_m_ under drought indicates the presence of enzyme isoforms different to those under optimal conditions (Kuzyakov and Blagodatskaya, 2015). This indication suggests a shift in microbial community structure and/or modification or shift of the enzyme system (released by roots, hyphae, or microorganisms) itself (Bradford, 2013) as an essential strategy to enhance drought tolerance. The difference in these enzyme-to-substrate affinities suggests conformational shifts at the root-soil and hyphae interface in the extracellular enzyme-substrate interactions. This could be a survival strategy employed by sorghum genotypes to enhance drought tolerance.

### 4.2 Uptake and allocation of mineral nitrogen by roots and mycorrhizal hyphae

As expected, a much higher proportion of the mineral ^15^N tracer was acquired from the mycorrhizosphere compared to the root-free mycosphere compartment (**Figures 3A, B**) This pattern was visible in shoot and root ^15^N recovery, confirming that mineral ^15^N, taken up by roots, is highly mobile in the plant biomass (White, 2011). Besides the key role of roots in taking up mineral N (Mäder et al., 2000; Miransari, 2011), there was a detectable contribution to the ^15^N uptake from the mycosphere irrespective of the moisture. This confirms that mycorrhizal hyphae also contributed to the ^15^N acquisition. This might have been attributed to the hyphae’s extremely narrow diameter, which enhances their ability to infiltrate very tiny pores and gain access to nutrient resources that are generally concealed from the roots’ direct access (Bahadur et al., 2019; Smith et al., 2010).

Mycorrhizal hyphae contributed a much lower degree of plant mineral N nutrition under well-watered conditions. Conversely, under drought, an enhanced contribution of ^15^N uptake by AMF hyphae from the mycosphere compartment was observed (**Figures 3A, B**). This contribution was between 4 to 11.5 times higher than in the well-watered plants. AMF can establish extensive hyphal networks, which tremendously increase the absorptive capacity of the roots and thus enhance water and nutrient uptake (Xiao et al., 2023). Our findings support observations by Kakouridis et al. (2022) that AMF hyphae may have the ability to bridge air gaps in soils to reach out for disconnected soil resources. We lack any imaging confirmation of such bridging of air gaps. However, for AMF to bridge the compartment between the inner and outer ring, filled with hydrophobic pearls with no water film on their surface, a bridging of air gaps to reach out for the other compartment seems unavoidable. The relatively higher contribution of the mycosphere to sorghum’s N nutrition under drought suggests that this ability of AMF may be highly relevant in supporting N acquisition during periods of water scarcity.

The higher affinity of AMF than of plant roots for the less mobile NH_4_^+^ (Pérez-Tienda et al., 2012) could also be a possible explanation for the increased N recovery under drought (Xiao et al., 2023). This holds not only true for mineral N, but AMF can also stimulate their mycosphere microbiome to secrete hydrolytic enzymes and transport via the hyphae the released monomers or mineralized N forms to the plant (Kaur and Suseela, 2020). The increased LAP activity of Gd’s mycosphere under drought (**Figure 5D**) suggests that this induction of protein mining by AMF seems to be a key drought adaptation strategy, at least for this genotype. In this study, we observed a far higher transport of the ^15^N applied to the mycosphere soil to the soil of the mycorrhizosphere compartment than we observed transport in the opposite direction, i.e. from the mycorrhizosphere to the mycosphere compartment (**Supplementary Figure S1**). This suggests that we did not observe a randomized N transport through filamentous microbiome members, just from one soil volume to another, but that our largely unidirectional transport can specifically be attributed to AMF, which allocate ^15^N from the outer ring to the plant compartment, and then also use it in the soil, e.g., for their own biomass or for the supportive hyphosphere microbiome.

The ability of filamentous soil microorganisms to bridge air gaps is particularly crucial with discontinuous water films in dry soils (Smith et al., 2010; Li et al., 2019) where AMF hyphae may still reach water-filled isolated soil micro-structures. In contrast, roots only have access to macro- and mesopores, which could result in shrinkage and occasional root death in the absence of moisture (Kakouridis et al., 2022). This strongly buttresses the significant role of AMF in N acquisition under moisture-limited conditions when mass flow and diffusion of ions are restricted (Tobar et al., 1994), and thus supports our hypothesis that AMF access N pools unavailable to roots under drought and convey it to the plants. In this context, it was rather astonishing that drought-stressed plants showed much lower AMF root colonization compared to the well-watered plants (**Table 1**)—a phenomenon already observed by Begum et al. (2019) and Neumann and George (2004). Nevertheless, water limitation strongly enhanced ^15^N acquisition, via the AMF pathway, as also observed in the studies of Gholamhoseini et al. (2013) and Symanczik et al. (2018), suggesting that mycorrhizal fungi colonization boosted N uptake under drought irrespective of the lower colonization rate.

The significant positive correlation between ^15^N uptake by sorghum genotypes from the mycosphere and the rate of AMF colonization under drought (**Figure 6F**) suggests that AMF hyphae might have played a role in facilitating the acquisition of N from the outer soil compartment. In contrast, under optimal conditions, the lack of correlation between ^15^N recovery and root colonization by AMF possibly indicates that high colonization under adequate moisture levels does not necessarily translate to high mycorrhizal N uptake or play a supportive role in plant nutrition. Whereas, under optimal conditions, the ^15^N uptake by AMF was very low for all sorghum genotypes, the genotypes displayed significant plasticity for ^15^N uptake under drought from the mycosphere. Under water-limited conditions, Gd showed the highest mineral ^15^N recovery in shoots and roots from the mycosphere (**Figures 3A, B**), suggesting that Gd’s N uptake might have resulted from an optimized activation and exploitation of its associated microbial partners, specifically AMF. The slightly higher mycorrhizal root colonization of Gd compared to the other genotypes under drought (**Table 1**) suggests that genotype-specific differences in colonization rates may serve as an indicator of AMF contribution to mineral nitrogen uptake (**Figure 3**).

### 4.3 Microbial utilization of plant-derived C

As already shown in *Zea mays* (Somasundaram et al., 2009)*, Festuca arundinacea* and *Medicago sativa* (Sanaullah et al., 2012), and *Trifolium repens* and *Lolium perene* (Qiao et al., 2022), a water deficit increases plant-derived C allocation into microbial biomass (**Figures 4A, B**). In response to restrictions in below-ground resource access, plants tend to direct a greater portion of their photosynthetic products directly to their roots or to their AMF partners, which are jointly in charge of obtaining the scarce resources—water and nutrients—under drought (Johnson, 2010).

Besides the rhizosphere microbiome and AMF, the AMF microbiome partners in the mycosphere profited strongly from the higher photoassimilate allocation below ground under drought (**Figure 4B**). Such an enhanced C transfer by the host plant to the AMF hyphae and its mycosphere microbiome very likely stimulates N mineralization in the soils and its transfer via AMF to the plant (Fellbaum et al., 2012; Kiers et al., 2011; Wang et al., 2021). It is feasible that a significant increase in assimilated ^13^C in the extractable microbial biomass of the mycosphere compartment could be a result of a direct transfer of plant-derived C to the AMF and their supporting hyphal microbiome (Kaiser et al., 2015; Kakouridis et al., 2024) to support resource acquisition under drought.

## 5 Conclusion

**Figure 7:**
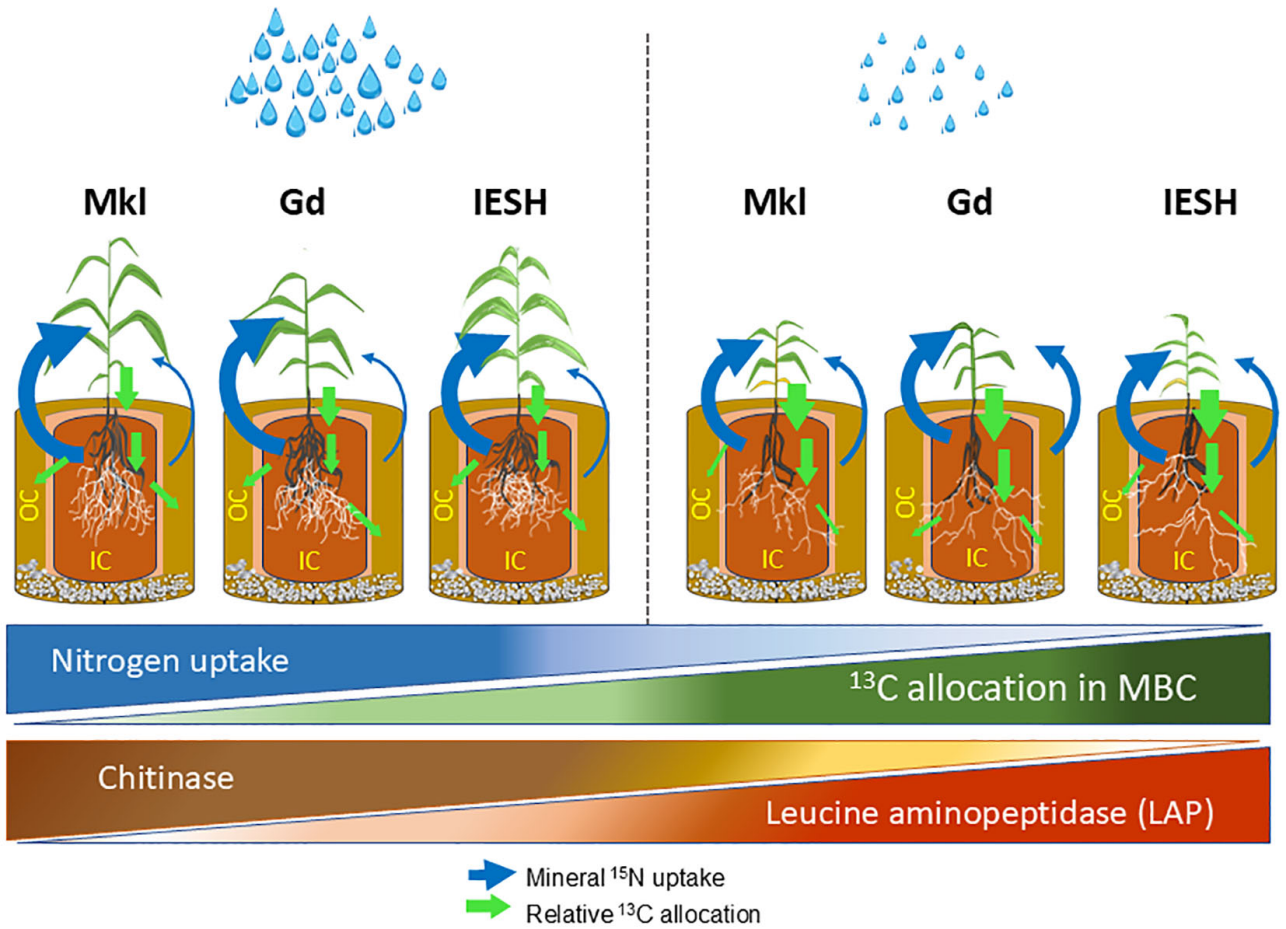
Schematic illustration of rhizosphere dynamics and growth performance of the three sorghum genotypes: Makueni local (Mkl), Gadam (Gd), and IESH 22012 (IESH) under optimal (left) and drought (right) conditions. The diagram depicts shoot and root biomass, mineral ^15^N uptake from the mycorrhizosphere (IC) and mycosphere (OC), relative ^13^C allocation in microbial biomass carbon (MBC), and the activity of enzymes [chitinase and leucine aminopeptidase (LAP)]. Variation in N uptake, ^13^C in MBC, and enzymatic activities are illustrated by triangular shapes. The triangles show the overall drought effects without consideration of individual genotypes, while the arrows indicate relative differences between the genotypes within each water treatment. Green arrows indicate carbon fluxes, while blue arrows represent mineral ^15^N fluxes, with arrow sizes corresponding to the relative quantities of ^13^C and mineral ^15^ N. Further details are explained in the main text.

To cope with the effects of water scarcity, plants adapt their enzymatic N mining activity in the rhizosphere. Maintaining constant chitinase activity in the mycorrhizosphere and additionally increasing LAP activity under drought may be an important strategy to overcome increasing N limitation under water scarcity. Thereby, genotype‐specific variations were observed in their responsiveness to AMF symbiosis and N acquisition following water limitation. Gd showed more pronounced N acquisition from the mycosphere under drought, potentially resulting from an enhanced interaction with AMF. However, there were no indications that modern sorghum breeding had negative effects on mycorrhizal growth receptiveness and/or the role of the tested N hydrolyzing enzymes for the N nutrition of sorghum. Irrespective of the genotype, we identified generic responses of sorghum’s N acquisition strategy to drought, which are strongly related to its interaction with the mycorrhizal partner. Overall, both AMF symbiosis and shifts in enzymatic N mining play a crucial role in sorghum’s N acquisition strategy, particularly under drought stress, by enhancing nutrient mobilization and availability in the rhizosphere (**Figure 7**).

Drought substantially reduced N uptake and AMF root colonization. However, the support of the mycorrhizal symbiosis in mineral N acquisition was enhanced for all sorghum genotypes under drought stress (**Figure 7**). Under optimal moisture content, sorghum genotypes use more C to form the above-ground biomass and N uptake largely occurred via the roots. However, in response to water limitation, there was a considerable shift toward investment into root C and a higher C supply for the below-ground microbial partners, the latter most likely to activate the AMF and the associated microbiome, and both jointly contribute to enhanced N mobilization and uptake. Therefore, we can conclude that enhanced C allocation below ground, and specifically to microbial biomass (including AMF), appears to be a key drought mitigation strategy of sorghum to cope with combined water and N limitation. AMF notably enhanced the mineral N uptake by the plant from the mycosphere, indicating their critical role in N acquisition from soil compartments not accessible by roots (Abdalla et al., 2023).

## Data Availability

The raw data supporting the conclusions of this article will be made available by the authors, without undue reservation.
