# Supplementary material for: Contribution of arbuscular mycorrhiza and exoenzymes to nitrogen acquisition of sorghum under drought
Source: Front Plant Sci. 2025 Apr 15;16:1514416. doi: 10.3389/fpls.2025.1514416 (PMC12037375; doi:10.3389/fpls.2025.1514416)
Supplement: Supplementary file 1 [file DataSheet1.docx]

**Supplementary materials**

**Table S1. Soil moisture content readings (%) in the inner and outer compartments from 4^th^ to 7^th^ week of growth**

|  | **6^th^ Oct** | | **11^th^ Oct** | | **15th Oct** | | **18^th^ Oct** | | **21^st^ Oct** | | **24^th^ Oct** | | **28^th^ Oct** | | **1^st^ Nov** | | **3rd Nov** | |
| --- | --- | --- | --- | --- | --- | --- | --- | --- | --- | --- | --- | --- | --- | --- | --- | --- | --- | --- |
|  | **Inner** | **Outer** | **Inner** | **Outer** | **Outer** | **Inner** | **Outer** | **Inner** | **Outer** | **Inner** | **Inner** | **Outer** | **Inner** | **Outer** | **Inner** | **Outer** | **Inner** | **Outer** |
| **Mkl** | 32.7 | 11.3 | 36.9 | 10.05 | 35.5 | 10.9 | 33.1 | 11.1 | 30.4 | 9.9 | 28.2 | 10.6 | 13.4 |  | 9.2 |  | 8.3 |  |
| **Gd** | 30.4 | 18,3 | 35.7 | 15.1 | 31.4 | 13.7 | 30.6 | 12.5 | 28.4 | 11.8 | 25.8 | 10.4 | 10.3 |  | 9.65 |  | 7.85 |  |
| **IESH** | 32.7 | 20,3 | 31.9 | 17,1 | 30,1 | 12.1 | 31.5 | 18.8 | 27.5 | 13.6 | 25.1 | 12.4 | 10.9 |  | 7.9 |  | 7.5 |  |

**Table S2. at% ^15^N in the shoot and root biomass labelled from inner and outer soil compartment under well-watered and drought conditions**

| **Water Availability** | **Genotypes** | **at.% ^15^N** | | | |
| --- | --- | --- | --- | --- | --- |
|  |  | **Shoot** | | **Root** | |
|  |  | Labelled soil compartment | | Labelled soil compartment | |
|  |  | Inner | Outer | Inner | Outer |
| Well-watered | Mkl | 1.29±0.19 | 0.36±0.004 | 1.48±0.17 | 0.39±0.005 |
|  | Gd | 1.28±0.21 | 0.36±0.006 | 1.41±0.11 | 0.40±0.01 |
|  | IESH | 1.32±0.03 | 0.37±0.0009 | 1.36±0.08 | 0.39±0.001 |
| Drought | Mkl | 0.78±0.090 | 0.42±0.05 | 0.91±0.05 | 0.46±0.085 |
|  | Gd | 0.99±0.046 | 0.61±0.12 | 0.98±0.08 | 0.70±0.19 |
|  | IESH | 0.97±0.092 | 0.44±0.56 | 0.98±0.1 | 0.54±0.154 |

Sorghum genotypes; Mkl - Makueni local, Gd - Gadam and IESH - IESH 2212
