# Supplementary material for: Contribution of arbuscular mycorrhiza and exoenzymes to nitrogen acquisition of sorghum under drought
Source: Front Plant Sci. 2025 Apr 15;16:1514416. doi: 10.3389/fpls.2025.1514416 (PMC12037375; doi:10.3389/fpls.2025.1514416)
Supplement: Supplementary file 2 [file DataSheet2.docx]

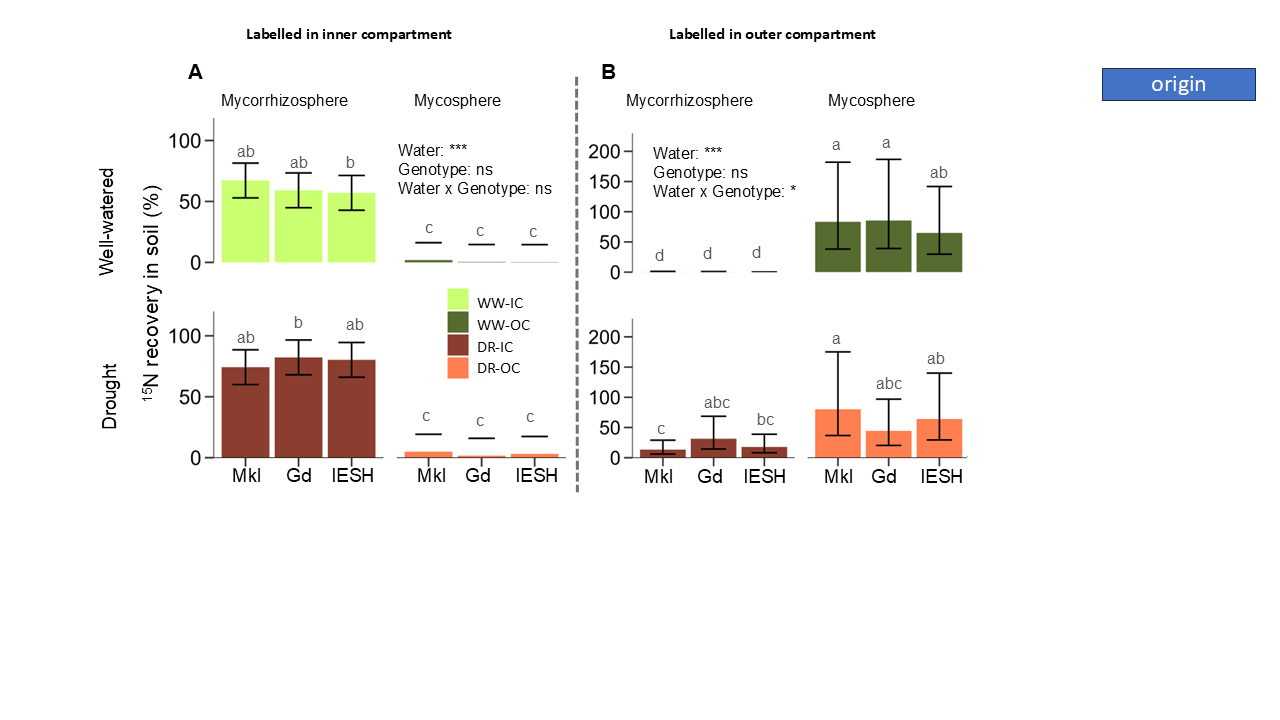


**Figure S1.** ¹⁵N tracer recovery in the mycorrhizosphere and mycosphere soil when labeled in the (**A**) inner compartment (IC) and (**B**) outer compartment (OC) under well-watered (green) and drought (brown) conditions. Data are presented as means ± SE (n = 4). Different letters indicate significant differences (p < 0.05). Sorghum genotypes: Mkl (Makueni local), Gd (Gadam), and IESH22012.
